# Mapping and visualization of global research progress on mitophagy in osteoarthritis: A bibliometric analysis (2008–2024)

**DOI:** 10.1097/MD.0000000000044421

**Published:** 2025-09-19

**Authors:** Liping Wang, Dengyan Bai, Xin Ma, Guan Wang, Zhangbin Luo

**Affiliations:** a Department of Anesthesia and Operation, Gansu Provincial Second People’s Hospital, Lanzhou City, Gansu Province, China; b Department of Orthopedics, Gansu Provincial Second People’s Hospital, Lanzhou City, Gansu Province, China.

**Keywords:** bibliometrics analysis, mitophagy, osteoarthritis

## Abstract

**Background:**

This study aimed to provide a thorough overview of research hotspots in mitophagy in osteoarthritis through a bibliometric analysis approach.

**Methods:**

A comprehensive literature search was conducted on the Web of Science Core Collection on September 9, 2024. Key metrics were calculated using Microsoft Excel 2019, and bibliometric analysis and visualization were performed with VOSviewer 1.6.20, CiteSpace 6.3.R1, and R 4.3.3.

**Results:**

The analysis covered 259 articles published between 2008 and 2024, involving 1679 authors from 924 institutions across 41 countries, indicating growing interest in mitophagy research related to osteoarthritis. Wenzhou Medical University emerged as the most prolific institution with 25 articles. The journal *Osteoarthritis and Cartilage* was a leading venue, showing high citation metrics and an impact factor of 7.2. The most cited article was “Pesticides and human chronic diseases: Evidence, mechanisms, and perspectives” from *Toxicology and Applied Pharmacology*. The keyword “apoptosis” was the most frequently used, along with significant terms like “oxidative stress” and “mitochondrial dysfunction.” Keyword burst analysis indicated intensified focus on “mammalian target of rapamycin” and “cell death.”

**Conclusion:**

This bibliometric analysis highlights a growing interest in mitophagy in osteoarthritis research, emphasizing its potential as a therapeutic target and signaling future advancements in this field.

## 1. Introduction

Osteoarthritis is a prevalent and chronic degenerative joint disease characterized by the progressive degradation of articular cartilage, resulting in symptoms such as pain, stiffness, swelling, and diminished mobility in affected joints.^[1]^ This deterioration is frequently accompanied by alterations in subchondral bone and synovial inflammation, which further exacerbate joint dysfunction.^[2]^ As a leading cause of disability among the elderly, osteoarthritis represents a significant global health burden, affecting millions worldwide and incurring substantial socio-economic costs, including lost productivity and increased healthcare expenditures.^[3]^ The management of osteoarthritis primarily focuses on symptom alleviation, which encompasses pain relief through pharmacological interventions (such as non-steroidal anti-inflammatory drugs), physical therapy, and, in severe cases, surgical interventions such as joint replacement.^[4]^ Although the etiology of osteoarthritis is complex and multifactorial – encompassing mechanical, genetic, and biochemical factors – recent studies have increasingly emphasized the role of mitochondrial dysfunction and cellular processes, such as autophagy, in the progression of the disease, thereby revealing potential therapeutic targets for mitigating cartilage degradation and reducing joint inflammation.^[5]^

Mitophagy, constitutes a critical process for maintaining cellular health by selectively degrading and recycling impaired mitochondria, which can accumulate in response to stress and aging.^[6]^ This process is particularly important in conditions like osteoarthritis, where mitochondrial dysfunction and autophagic imbalance contribute to disease progression.^[7]^ In human chondrocytes, defects in autophagic pathways exacerbate mitochondrial dysfunction, contributing to oxidative stress and cell death.^[8]^ Research on post-traumatic osteoarthritis has highlighted the function of autophagy in mitigating inflammation, sustaining chondrocyte homeostasis, and postponing the degeneration of joint tissues.^[9]^ Furthermore, studies have indicated that autophagic flux – referring to the comprehensive process of autophagy from induction to degradation – is frequently compromised in osteoarthritis chondrocytes, suggesting that not only the initiation of mitophagy but also the completion of the degradation process may be hindered.^[10]^ Given its pivotal role in cellular maintenance and the prevention of cartilage degradation, mitophagy emerges as a promising area of research for the development of interventions aimed at treating or managing osteoarthritis.

Bibliometrics is a quantitative methodology for analyzing scientific publications that provides valuable insights into the impact, trends, and advancements within a specific field.^[11]^ This approach aids in uncovering emerging topics, recognizing influential works, and supporting decision-making processes related to research funding and policy development within the scientific community.^[12]^ Joshi et al conducted a bibliometric analysis of knee osteoarthritis research, highlighting key topics such as phonophoresis, Otago exercises, and the star excursion balance test, while emphasizing the significance of exercise therapy and suggesting future directions that include comprehensive evaluations of research policies and enhanced international collaboration.^[13]^ To date, there has been no dedicated bibliometric analysis on mitophagy in osteoarthritis. Therefore, this study aims to conduct a thorough bibliometric analysis of research trends regarding mitophagy in osteoarthritis to identify key trends, influential studies, and potential future directions in this emerging area.

## 2. Methods

### 2.1. Search strategies and data source

The Web of Science Core Collection (WoSCC) was employed to conduct a literature search from 2008 to 2024. Recognized as a comprehensive and authoritative database, the WoSCC provides access to high quality academic publications across diverse disciplines.^[14]^ The search formula utilized was: (((TS = (osteoarthritis)) OR TS = (osteoarthritis)) OR TS = (osteoarthrosis)) OR TS = (arthritis) AND (TS = (mitochondrial autophagy)) OR TS = (mitophagy). Only English-language publications were included, with a specific focus on articles among various document types. To minimize discrepancies resulting from database updates, literature retrieval was conducted on September 9, 2024. The screening process was undertaken by 2 independent reviewers, with any disagreements resolved through discussions to achieve consensus.

### 2.2. Statistical analysis and visualization

Three bibliometric tools were utilized for visualization and comprehensive analysis of academic data: VOSviewer 1.6.20, CiteSpace 6.3.R1, and R 4.3.3. VOSviewer, noted for its versatility, was crucial in mapping institutional and author collaborations, co-authorship, citation patterns, keyword co-occurrence networks, and co-citation networks.^[15]^ This tool facilitated the visualization and analysis of complex collaborative networks within academia. CiteSpace was employed to identify keyword bursts, facilitating an improved understanding of emerging trends and research hotspots. The parameters were established as follows: time slicing was defined from January 2008 to September 2024, with keywords selected as the node type. A keyword node threshold of 5 was applied prior to each fragment, and pruning was executed utilizing the pathfinder and clip merge network methods. Visualization analysis was conducted based on these parameters to generate a keyword timeline within the research field of mitophagy in osteoarthritis. The R 4.3.3 (https://www.bibliometrix.org) was utilized for a thorough bibliometric analysis. This tool played a pivotal role in analyzing and mapping the global distribution of research output, uncovering significant trends and patterns, and assessing the impact of authors, journals, and institutions within the dataset. The H-index quantified the academic impact of individuals and journals, serving as a balanced measure of scholarly influence.^[16,17]^ Journal citation reports (JCR) quartiles and the impact factor (IF) were utilized to evaluate journal prestige and citation influence. JCR quartiles classify journals into 4 tiers, with Q1 indicating the highest academic impact, while the IF measures the average citations received by a journal’s articles over the preceding 2 years. The most recent 2024 JCR and IF data were employed to ensure an up-to-date assessment of journal prestige and citation influence.

## 3. Results

### 3.1. Overview of global research on mitophagy in osteoarthritis

**Figure 1.**
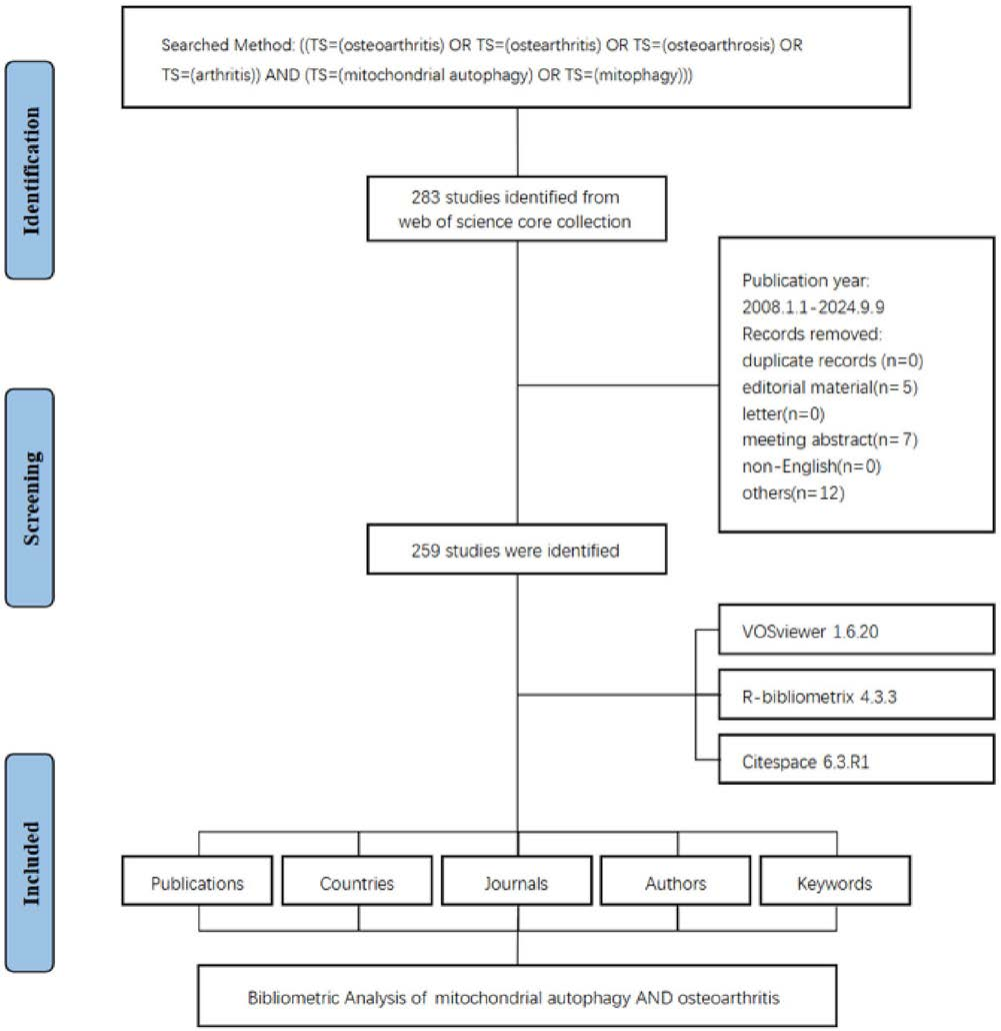
Flowchart of the literature screening process.

**Figure 2.**
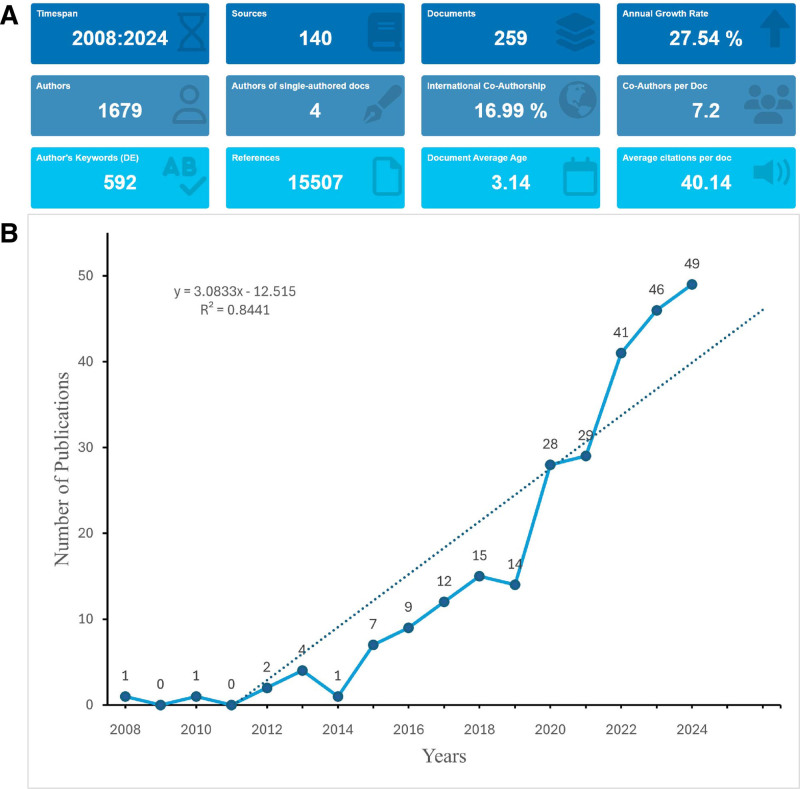
Overview of mitophagy in osteoarthritis. (A) Comprehensive overview of the bibliometric analysis. (B) Annual growth of publications on osteoarthritis.

The data screening process is depicted in Figure 1. The analysis revealed that 1679 authors from 924 distinct institutions across 41 countries and regions contributed to the 259 publications included in this study. These articles were disseminated across 140 different journals and collectively cited a total of 15,507 references. The annual number of publications pertaining to mitophagy in osteoarthritis research had exhibited a clear upward trend over the years (Fig. 2A). We applied a linear growth model with the equation: *y* = 3.0833*x* − 12.515, resulting in an 𝑅^2^ value of 0.8441. This indicated a strong correlation between the number of publications and the years, reflecting an upward trend in research interest over time. Beginning with only a few publications around 2008, the field began to gain traction in the early 2010s, characterized by a gradual increase in the number of studies published each year. From 2016 onward, there was a notable acceleration in publication activity, signifying growing interest and advancements in this area. The period from 2020 to 2024 experienced a particularly significant increase, with the number of publications reaching 49 in 2024. This upward trajectory suggested that research on mitophagy in osteoarthritis is likely to continue expanding, thereby attracting sustained interest in the future (Fig. 2B).

Among the most cited studies in this field was “*Pesticides and human chronic diseases: evidences, mechanisms, and perspectives*,” published in 2013 in *Toxicology and Applied Pharmacology* (IF = 3.3). This influential article has garnered a total of 760 citations and provides comprehensive insights into the associations between pesticide exposure and chronic human diseases.^[18]^ Another highly cited work was “Aging and the pathogenesis of osteoarthritis,” published in 2016 in Nature Reviews Rheumatology (IF = 29.4), which has accumulated 753 citations. This review examined the relationship between aging and the progression of osteoarthritis, contributing significantly to the field.^[19]^ This review examined the relationship between aging and the progression of osteoarthritis, contributing significantly to the field. Additionally, the third most cited article was “*Age-related mitochondrial dysfunction and oxidative stress in osteoarthritis*,” published in 2014 in Annals of the Rheumatic Diseases (IF = 16.1), which has accumulated 742 citations. This research investigated the role of mitochondrial dysfunction in osteoarthritis, elucidating the underlying mechanisms of the disease.^[20]^

### 3.2. Global distribution of research by country

**Figure 3.**
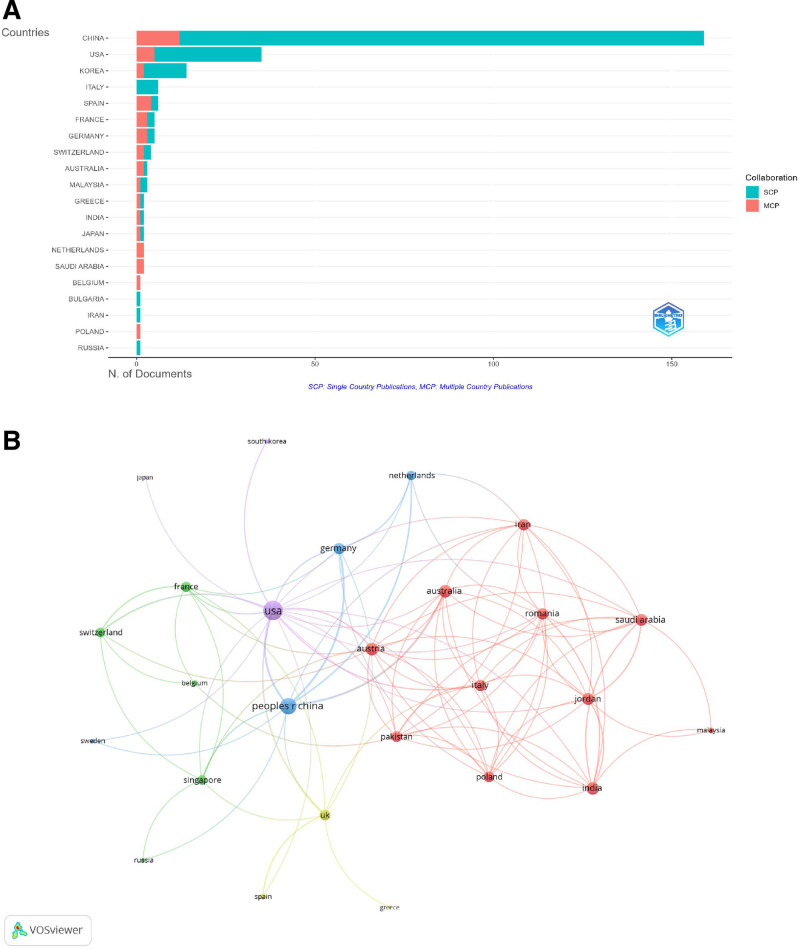
Global distribution and collaboration of mitophagy in osteoarthritis. (A) Distribution of corresponding author’s publications by country. (B) Visualization map depicting the collaboration among different countries. MCP = multiple country publications, SCP = single country publications.

China was at the forefront of research on mitophagy in osteoarthritis, with a total of 159 publications. China ranked first in total publications (TP) (557) and total citations (TC) (3501), although it exhibited a relatively lower multiple country publications (MCP) ratio of 0.075, indicating a predominant focus on single-country publications. The United States followed with 35 publications, ranking second in both TP (116) and TC (3187). The USA also demonstrated a higher MCP ratio of 0.143, reflecting a greater level of international collaboration compared to China. Korea ranked third, with 14 publications and a similar citation count (988), exhibiting an MCP ratio of 0.143. Italy and Spain each had 6 publications, ranking fourth and fifth in TP, respectively, with notable MCP ratios Spain, in particular, possesses a ratio of 0.667, suggesting significant international collaborations. Other countries, such as Switzerland and Australia, also display high MCP ratios (0.500 and 0.667, respectively), indicating robust international research partnerships. Conversely, countries like Iran, despite having fewer publications, demonstrated a high average citation per publication (760 citations), underscoring the impact of their limited research output. The varying MCP ratios among countries reflected diverse approaches to international collaboration within this research domain (Fig. 3A, Table 1). The visualization map illustrating international collaborations among countries in mitophagy research related to osteoarthritis was presented. The USA was identified as having the highest number of collaborations, with a total link strength of 34. China followed closely, with a total link strength of 24. Australia also exhibited a notable presence, with a total link strength of 15 (Fig. 3B).

**Table 1:** Publication and citation profiles of leading countries.

Country	Articles	Freq	MCP_ratio	TP	TP_rank	TC	TC_rank	Average citations
China	159	0.614	0.075	557	1	3501	1	22
USA	35	0.135	0.143	116	2	3187	2	91.1
Korea	14	0.054	0.143	58	3	988	3	70.6
Italy	6	0.023	0.000	29	4	209	7	34.8
Spain	6	0.023	0.667	27	5	158	10	26.3
France	5	0.019	0.600	22	6	172	9	34.4
Germany	5	0.019	0.600	12	9	196	8	39.2
Switzerland	4	0.015	0.500	9	11	215	6	53.8
Australia	3	0.012	0.667	13	8	81	14	27
Malaysia	3	0.012	0.333	7	12	77	15	25.7
Greece	2	0.008	0.500	5	15	48	18	24
India	2	0.008	0.500	14	7	38	19	19
Japan	2	0.008	0.500	5	16	73	16	36.5
Netherlands	2	0.008	1.000	7	13	88	13	44
Saudi Arabia	2	0.008	1.000	3	26	95	12	47.5
Belgium	1	0.004	1.000	4	18	5	21	5
Bulgaria	1	0.004	0.000	4	19	0	23	0
Iran	1	0.004	0.000	4	20	760	4	760
Poland	1	0.004	1.000	3	24	112	11	112
Russia	1	0.004	0.000	3	25	4	22	4

Articles refer to the publications of corresponding authors only; average citations are the average number of citations per publication.

Freq = frequence of total publications, MCP_ratio = proportion of multiple country publications, TP = total publications, TP_rank = rank of total publications, TC = total citations, TC_rank = rank of total citations.

### 3.3. Institutional influence and contributions analysis

**Figure 4.**
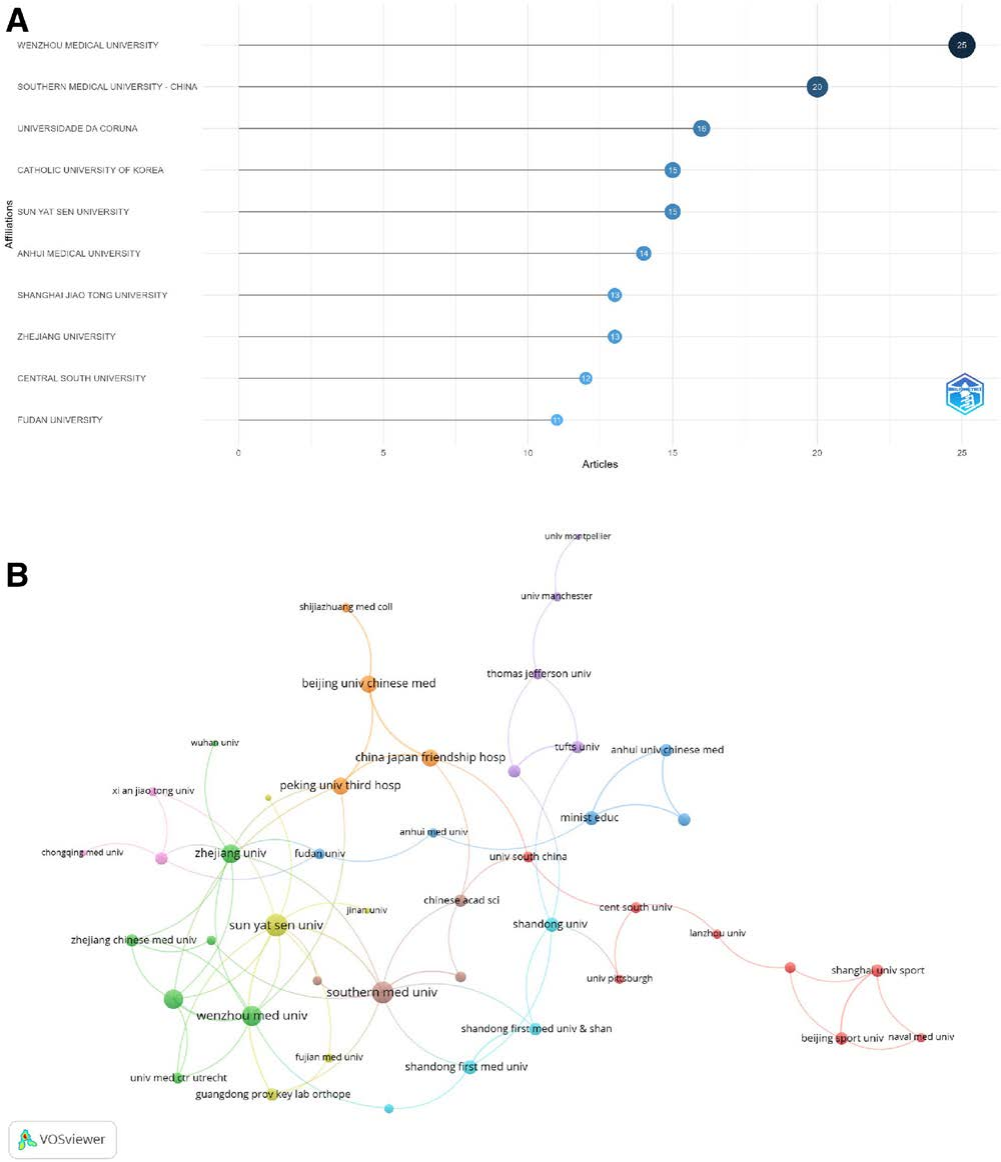
Institutional contributions and collaborations of mitophagy in osteoarthritis. (A) Top 10 institutions by article count and rank. (B) Visualization map depicting the collaboration among different institutions.

The 10 leading institutions in mitophagy research pertaining to osteoarthritis are depicted in Figure 4A. Wenzhou Medical University was at the forefront, with 25 published articles, underscoring its substantial contributions to this field. Southern Medical University in China occupied the second position, with 20 articles, reflecting its active engagement in the advancement of knowledge in this area. Universidade da Coruña follows with 16 articles, indicating its significant involvement in the research. Figure 4B illustrates the collaborative interactions among 45 institutions engaged in international partnerships, each contributing a minimum of 2 published articles in mitophagy research relevant to osteoarthritis. Sun Yat Sen University was notable for exhibiting the highest total link strength of 12, suggesting a robust collaborative network in this research domain. Southern Medical University closely trailed with a total link strength of 11, demonstrating its critical role in fostering research partnerships. Chang Gung University also exhibited strong international collaboration, with a link strength of 10, highlighting its influence and connectivity within the global research community.

### 3.4. Journal analysis

**Figure 5.**
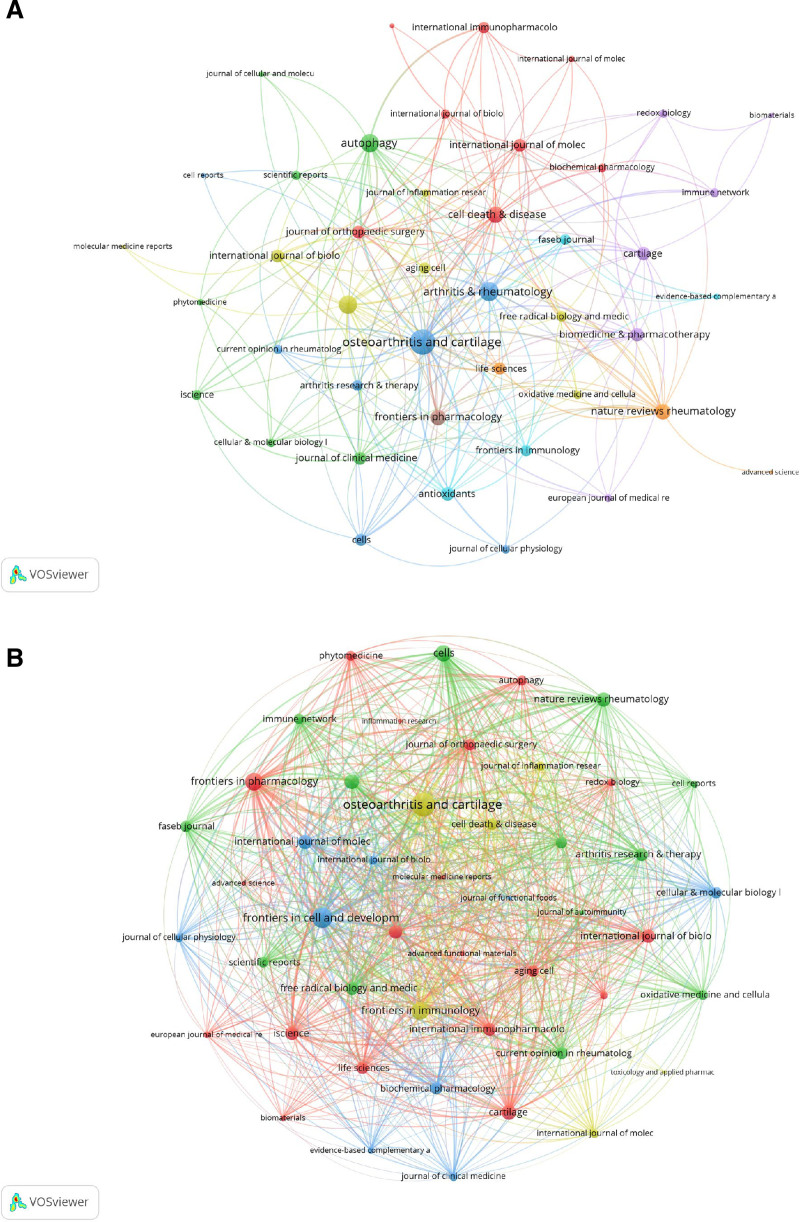
Network analyses of journals of mitophagy in osteoarthritis. (A) Journal co-occurrence network diagram. (B) Journal bibliography coupling network diagram.

A comprehensive overview of the top 20 most productive journals in the domain of mitophagy in osteoarthritis was presented (Table 2). “*Osteoarthritis and Cartilage*” is identified as the preeminent journal in this field, exhibiting an H-index of 9, an IF of 7.2, and a total of 13 publications. This positions it first in both TP and TC (710). The “*International Journal of Molecular Sciences*” ranked second in TP with 9, though it is ranked ninth in TC (303), indicating a robust presence within the discipline. *Cell Death & Disease* was positioned seventh in TP with 5, distinguished by its high IF (IF = 8.1), while it holds the 16th position in TC (186). The co-occurrence network of journals associated with mitophagy research in osteoarthritis (Fig. 5A). The 3 principal journals with the highest total link strength in this network were “*Osteoarthritis and Cartilage*” (71), “*Arthritis & Rheumatology*” (40), and “*Autophagy*” (37). Figure 5B displays the journal coupling network diagram, wherein the 3 journals with the highest total link strength were “*Osteoarthritis and Cartilage*” (1557), “*Frontiers in Cell and Developmental Biology*” (1116), and “Frontiers in Pharmacology” (856).

**Table 2:** Top 20 most productive journals in the field of mitophagy in osteoarthritis.

Journal	H_index	IF_2023	JCR_quartile	PY_start	TP	TP_rank	TC	TC_rank
Osteoarthritis and Cartilage	9	7.2	Q1	2012	13	1	710	1
International Journal of Molecular Sciences	6	4.9	Q2	2015	9	2	303	9
Cell death & Disease	5	8.1	Q1	2017	5	7	186	16
Frontiers in Immunology	5	5.7	Q1	2016	7	4	145	20
Frontiers in Pharmacology	5	4.4	Q1	2022	8	3	131	27
Phytomedicine	5	6.7	Q1	2013	7	5	44	92
Arthritis & Rheumatology	4	11.4	Q1	2015	4	9	234	11
Current Opinion in Rheumatology	4	5.2	Q1	2017	4	13	54	72
Free Radical Biology and Medicine	4	7.1	Q1	2018	4	14	201	15
Frontiers in Cell and Developmental Biology	4	4.6	Q1	2020	5	8	92	40
Journal of Orthopedic Surgery and Research	4	2.8	Q1	2020	4	17	21	185
Scientific Reports	4	3.8	Q1	2016	4	18	204	14
Arthritis Research & Therapy	3	4.4	Q1	2015	4	10	211	12
Autophagy	3	14.6	Q1	2016	3	20	329	7
Cartilage	3	2.7	Q1	2021	3	21	33	118
Cell Reports	3	7.5	Q1	2018	3	22	74	53
Cells	3	5.1	Q2	2019	4	12	79	48
International Journal of Molecular Medicine	3	5.7	Q1	2019	3	23	64	60
Journal of Cellular Physiology	3	4.5	Q1	2019	4	15	109	35
Journal of Inflammation Research	3	4.2	Q2	2021	4	16	18	207

H_index is the h-index of the journal, which measures both the productivity and citation impact of the publications, IF is the impact factor, indicating the average number of citations to recent articles published in the journal, JCR_quartile is the quartile ranking of the journal in the journal citation reports, indicating the journal’s ranking relative to others in the same field (Q1: top 25%, Q2: 25%–50%, Q3: 50%–75%, Q4: bottom 25%), average citations are the average number of citations per publication.

TP = total publications, TP_rank = rank of total publications, TC = total citations, TC_rank = rank of total citations, PY_start = publication year start (indicating the year the journal started publication).

### 3.5. Authorship contributions analysis

The following was a list of the top 20 influential authors in the field of mitophagy research related to osteoarthritis. Jorg J. Goronzy leaded this cohort with an H-index of 5, a g-index of 5, and a high m-index of 0.83, reflecting his significant impact since he commenced his contributions in 2019. He ranked second in TP (5) and sixth in TC (248). Cornelia M. Weyand possessed a comparable profile, with an h-index and g-index of 5, an m-index of 0.83, and also 248 citations, resulting in her fourth position in citation rank. Francisco J. Blanco had also made notable contributions, evidenced by an h-index of 4, a g-index of 5, and a moderate m-index of 0.40 since 2015. He ranked first in total publication fraction (0.86) and 22nd in TC (158). Liu-Bryan, Ru, and Terkeltaub, Robert each had an h-index of 4 and are tied for second place in TC (702), underscoring their significant contributions to this research domain (Table 3).

**Table 3:** Publication and citation profiles of high-impact authors.

Authors	H_index	g-index	m-index	PY_start	TP	TP_Frac	TP_rank	TC	TC_rank
Goronzy Jorg J.	5	5	0.83	2019	5	1.42	2	248	6
Weyand Cornelia M.	5	5	0.83	2019	5	1.42	4	248	6
Blanco Francisco J.	4	5	0.40	2015	5	0.86	1	158	22
Jin Eun-Jung	4	4	0.40	2015	4	0.69	5	70	28
Liu-Bryan Ru	4	4	0.40	2015	4	1.12	6	702	2
Song Jinsoo	4	4	0.40	2015	4	0.69	7	70	28
Terkeltaub Robert	4	4	0.40	2015	4	1.12	8	702	2
Borzi Rosa Maria	3	3	0.33	2016	3	0.47	10	175	13
Cetrullo Silvia	3	3	0.33	2016	3	0.47	12	175	13
Chen Xiaoyu	3	3	0.33	2016	3	0.54	14	73	26
Cho Mi-La	3	3	0.38	2017	3	0.29	15	165	17
D’adamo Stefania	3	3	0.33	2016	3	0.47	18	175	13
Flamigni Flavio	3	3	0.33	2016	3	0.47	20	175	13
He Yuzhe	3	3	0.60	2020	3	0.39	22	160	19
Lotz Martin	3	3	0.30	2015	3	0.59	24	278	4
Lotz Martin K.	3	3	0.30	2015	3	0.54	25	262	5
Park Sung-Hwan	3	3	0.38	2017	3	0.29	28	165	17
Rego-Perez Ignacio	3	4	0.75	2021	5	0.78	3	24	37
Vaamonde-Garcia Carlos	3	3	0.75	2021	3	0.28	30	12	42
Wang Gaoyuan	3	3	0.33	2016	3	0.54	31	73	26

H_index is the h-index of the journal, which measures both the productivity and citation impact of the publications, g_index is the g-index of the journal, which gives more weight to highly-cited articles, m_index is the m-index of the journal, which is the h-index divided by the number of years since the first published paper, average citations are the average number of citations per publication,

TP = total publications, TP_rank = rank of total publications, TC = total citations, TC_rank = rank of total citations, PY_start = publication year start (indicating the year the journal started publication).

**Figure 6.**
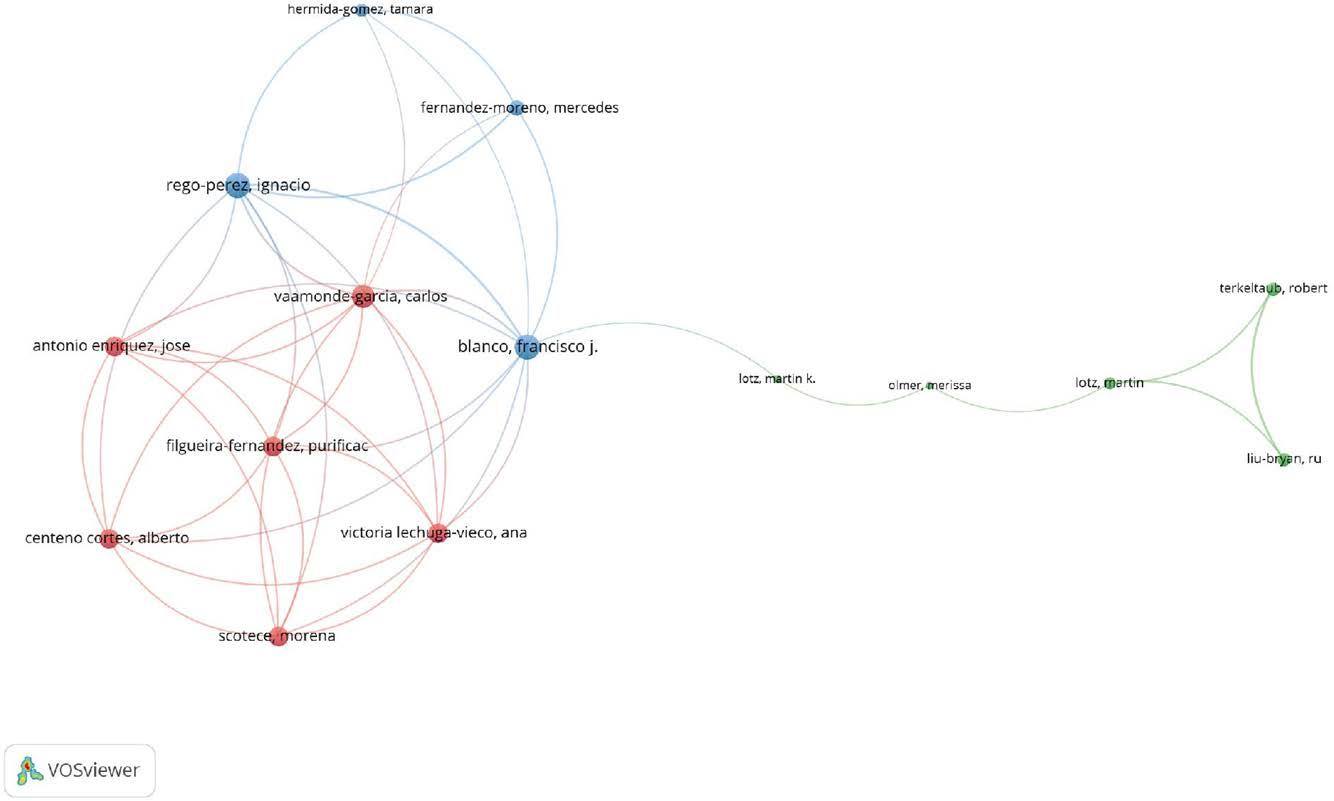
Visualization map depicting the collaboration among different authors.

The collaboration network comprised 15 authors who have participated in international partnerships, each having authored a minimum of 2 articles centered on mitophagy research in osteoarthritis. Ignacio Rego-Perez emerged as the most collaborative author, exhibiting a total link strength of 22, which indicated a robust and extensive network of international collaborations. Francisco J. Blanco closely followed, with a link strength of 21, reflecting his considerable collaborative contributions to the research community. Carlos Vaamonde Garcia also demonstrated significant collaborative engagement, with a total link strength of 18, highlighting his active involvement in promoting international research connections (Fig. 6).

### 3.6. Keyword co-occurrence and emerging research frontiers

A comprehensive keyword analysis of the selected articles identified 145 keywords, each occurring a minimum of 3 times. The primary keywords associated with mitophagy in osteoarthritis, the term “apoptosis” was particularly prominent, appearing 51 times with a total link strength of 283. Other significant keywords include “oxidative stress” and “activation,” underscoring essential processes in osteoarthritis research. Additionally, keywords such as “mitochondrial dysfunction” (total link strength of 161) and “articular cartilage” (total link strength of 127) emphasized the focus on mechanisms that influence cartilage health in osteoarthritis studies (Table 4).

**Table 4:** Co-occurrence analysis of top 20 keywords.

Keyword	Occurrences	Total link strength
Autophagy	72	367
Apoptosis	51	283
Oxidative stress	46	244
Expression	33	184
Activation	38	184
Osteoarthritis	34	181
Mechanisms	29	168
Mitochondrial dysfunction	28	161
Cartilage	26	152
Articular-cartilage	25	127
Cells	25	119
Mitophagy	24	114
Chondrocytes	18	113
Dysfunction	19	111
Pathogenesis	22	111
Inhibition	19	110
Disease	20	100
Cellular senescence	16	99
Cell-death	18	93
Inflammation	19	90

**Figure 7.**
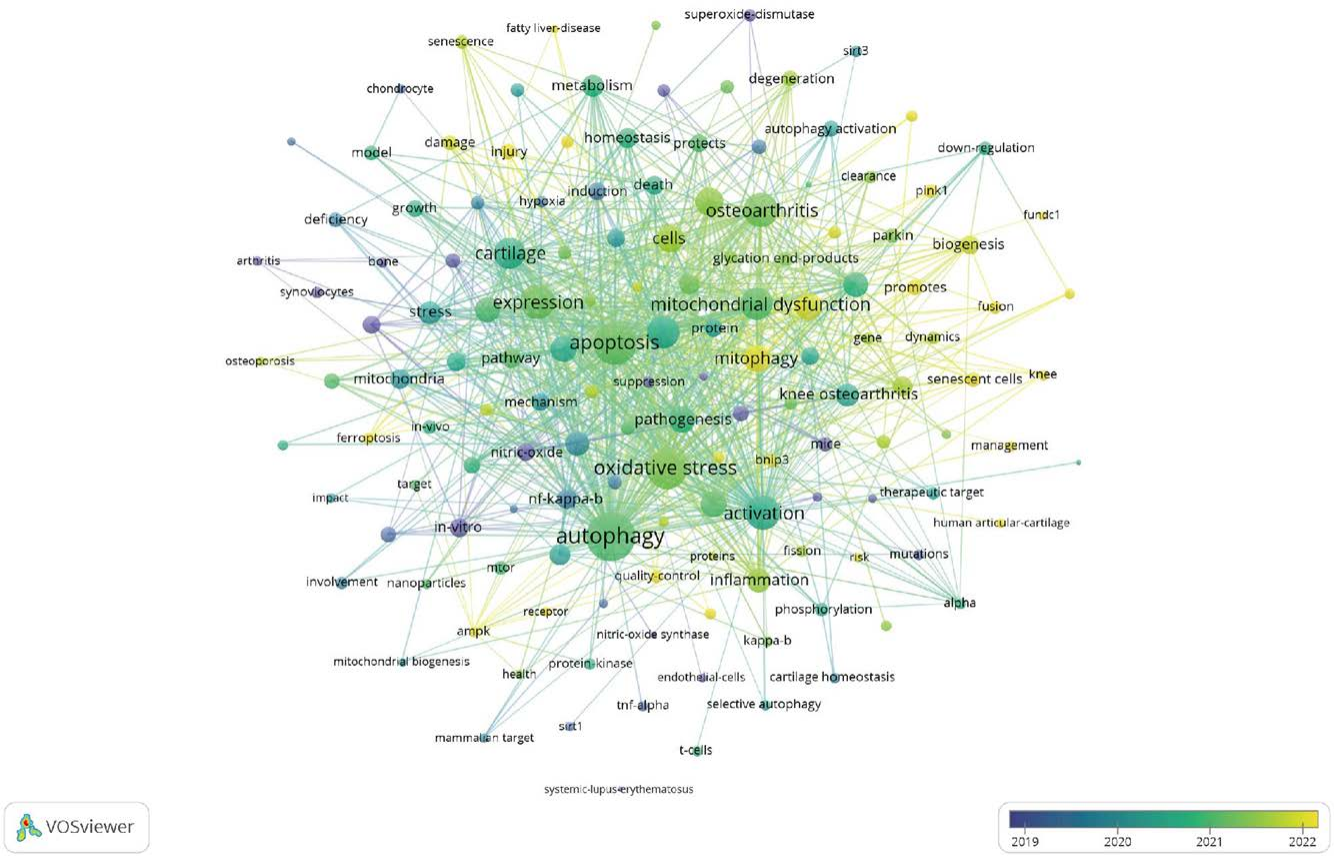
Visual analysis of keyword co-occurrence network analysis.

**Figure 8.**
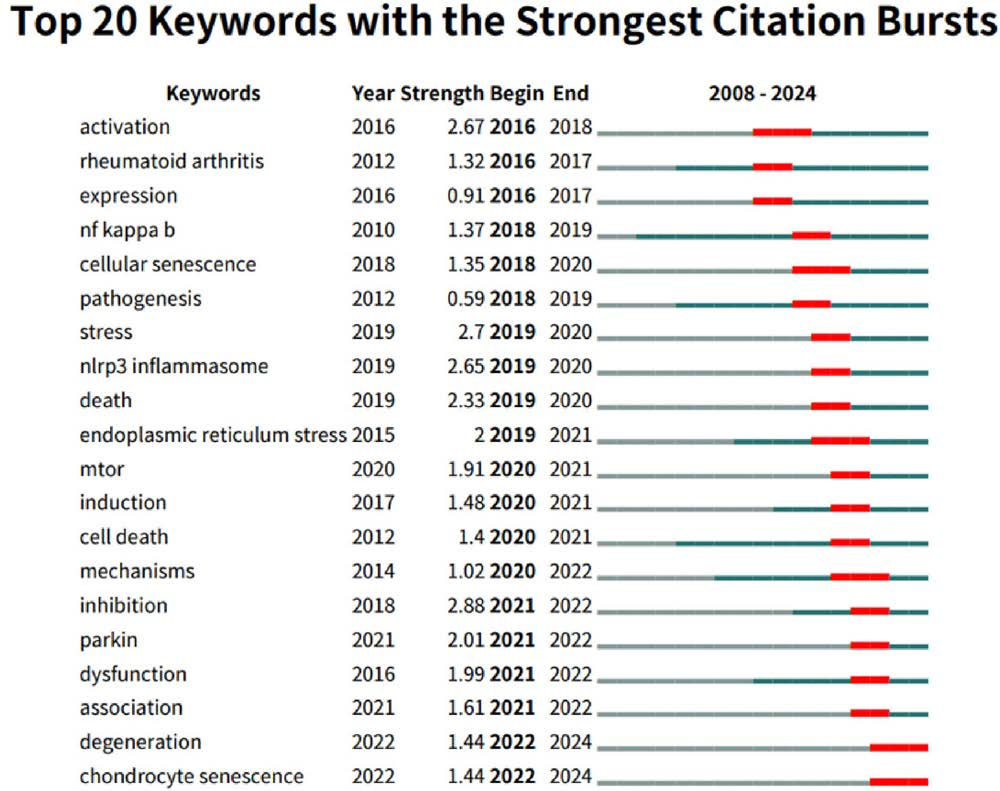
Top 20 keywords with the strongest citation bursts.

The visual analysis of the keyword co-occurrence network in mitophagy research related to osteoarthritis was present, the different colors indicated in the network represent the average publication year of various keywords. A total of 145 keywords with a minimum of 3 occurrences were identified. The shift from green (such as “mitochondrial dysfunction” and “oxidative stress”) to yellow (like “biogenesis” and “senescence”) illustrated the evolving research focus over time. More recent keywords, including “biogenesis,” “fusion,” and “senescence,” are shown in yellow, representing their emergence in studies published around 2021 to 2022 (Fig. 7). A burst analysis of keywords revealed evolving trends in mitophagy research related to osteoarthritis (Fig. 8). The keyword “inhibition” experienced the most significant citation burst, reflecting a recent and growing interest in therapeutic interventions targeting inhibitory pathways. Since 2020, other keywords have shown notable bursts, indicating key areas of focus: “mTOR” and “induction” highlight the exploration of cellular growth and regulatory mechanisms; “cell death” underscores interest in apoptosis and related processes in osteoarthritis. Additionally, keywords such as “mechanisms,” “parkin,” “association,” and “dysfunction” signaled a recent concentration on mitochondrial health and its implications for osteoarthritis. The terms “degeneration” and “chondrocyte senescence” indicated a sustained interest in understanding cartilage degradation and cellular aging.

## 4. Discussion

### 4.1. General information

This study analyzed 259 publications from 2008 to 2024. Research on mitophagy in osteoarthritis has shown a consistent upward trend since 2008, indicating a growing interest in mitochondrial dysfunction, oxidative stress, and their roles in osteoarthritis pathogenesis. China, particularly through institutions such as Wenzhou Medical University, has emerged as a leading contributor to research on mitophagy in osteoarthritis.^[21]^ This prominence reflects China’s robust biomedical research infrastructure and the substantial population of osteoarthritis patients in the country, creating considerable demand for research in this area.^[22]^ China’s collaborative research culture, integrating universities, hospitals, and research centers with advanced technologies like bioinformatics and molecular biology, has driven progress in osteoarthritis treatment, exemplified by Wenzhou Medical University’s efforts, solidifying China’s pivotal role in the field.^[23]^

In terms of authorship, Francisco J. Blanco stands out as one of the most prolific contributors. His team focuses on mitochondrial dysfunction and oxidative stress, which is critical for understanding the intricate cellular mechanisms underlying osteoarthritis. For example, Blanco et al demonstrates that diabetes exacerbates cartilage damage in osteoarthritis in mouse models and activating autophagy through inhibition of mammalian target of rapamycin (mTOR) effectively reduces cartilage degradation and synovial inflammation. This finding highlighted a specific cellular pathway that may be targeted for therapeutic intervention.^[24]^

### 4.2. Emerging research hotspots

A significant area of research in osteoarthritis focuses on cellular processes related to the disease, highlighting the critical roles of “apoptosis” and “oxidative stress” in osteoarthritis progression. Oxidative stress impairs mitochondrial function and disrupts autophagy, particularly in chondrocytes affected by osteoarthritis.^[25]^ This dysfunction is linked to increased mitochondrial damage and subsequent cellular death. Understanding mitochondrial health and dysfunction has become central to uncovering the cellular mechanisms underlying osteoarthritis, with related terms such as “mitochondrial dysfunction” and “biogenesis” gaining prominence. Research suggests that activating autophagy can protect human chondrocytes from mitochondrial dysfunction, highlighting its potential as a therapeutic target in osteoarthritis.^[26]^ Additionally, irisin, a myokine, has been found to upregulate mitochondrial biogenesis in chondrocytes, improving mitochondrial membrane potential and reducing oxidative damage. This protective mechanism is crucial for slowing osteoarthritis progression.^[27]^ This underscores an expand, underscoring a growing body of research investigating the therapeutic benefits of promoting mitochondrial health, particularly through mechanisms like mitophagy, in the context of osteoarthritis.

Inflammation represents a critical area of investigation in osteoarthritis research, particularly in relation to its association with cartilage degradation. Terminology such as “cartilage,” “articular cartilage,” and “inflammation” underscores the imperative to explore the role of inflammation in the pathophysiology of osteoarthritis. The autophagic protein DEPP is integral to the regulation of mitophagy in response to pro-inflammatory cytokines, a process that is essential for preserving chondrocyte viability.^[28]^ Inflammatory responses in the joint environment can lead to cartilage tissue degradation, underscoring the significance of inflammation in osteoarthritis.

### 4.3. Emerging research trends

Keyword burst analysis reveals trends and primary research interests in mitophagy in osteoarthritis over time. From 2020 to 2021, terms like “mTOR” and “cell death” highlight autophagy and apoptosis pathways, as research increasingly aims to uncover the cellular mechanisms influencing joint health. Inhibition of the phosphatidylinositol 3-kinase/AKT/mTOR pathway has been shown to enhance autophagy in osteoarthritis chondrocytes, leading to reduced inflammation and protection against cartilage degradation.^[29]^ Additionally, overexpression of sirtuin 3 can alleviate osteoarthritis symptoms by modulating the mTOR pathway, boosting autophagy while lowering apoptosis and inflammation.^[30]^ However, excessive autophagy may also contribute to cell death in cartilage affected by osteoarthritis,^[31]^ highlighting its context-dependent dual effects.

From 2021 to 2022, keywords such as “parkin,” “dysfunction,” and “inhibition” indicate a growing interest in mitochondrial quality in osteoarthritis. Parkin is crucial for removing damaged mitochondria in osteoarthritis chondrocytes, where it helps regulating reactive oxygen species levels and prevents apoptosis. This function is essential for maintaining chondrocyte viability under stress.^[32]^ Research involving 3-methyladenine, an autophagy inhibitor, has shown that inhibiting autophagy worsens cartilage degeneration in osteoarthritis. In experimental models, reduced autophagy has been linked to greater chondrocyte damage and cartilage loss, highlighting the protective role of autophagy in osteoarthritis.^[33]^ These findings emphasize the need to maintain mitochondrial integrity to preserve joint health.

From 2022 to 2024, the emergence of keywords like “degeneration” and “chondrocyte senescence” reflects recent interest in the degenerative processes associated with osteoarthritis, particularly regarding how cellular senescence affects chondrocytes. Aged chondrocytes increasingly exhibit a senescence-associated secretory phenotype, characterized by heightened inflammatory responses and oxidative stress. Enhancing autophagy may help mitigate these effects, potentially slowing osteoarthritis progression.^[34]^ Studies have shown that interventions aimed at boosting autophagy can reduce cellular senescence in osteoarthritis, thereby promoting chondrocyte survival and function. For example, fibroblast growth factor 21 has been shown to decrease senescence and apoptosis in osteoarthritis chondrocytes by activating autophagy.^[35]^ To reveal the intersection of aging and osteoarthritis, future study should focus on cartilage-specific degeneration mechanisms.

In summary, several key areas may emerge as focal points for future studies. First, targeted mitochondrial therapies could become more prominent, aiming to clear damaged mitochondria in chondrocytes and reduce oxidative stress, potentially slowing osteoarthritis progression. Second, future research is likely to investigate how regulating autophagy can be used to diminish inflammatory processes, offering potential strategies for protecting joint health. Lastly, the integration of precision medicine approaches tailored to individual mitochondrial dysfunctions may refine treatment protocols, allowing for more personalized and effective therapies.

## 5. Limitations

This study presents several limitations. Firstly, the analysis relied exclusively on the WoSCC, which might exclude relevant studies from other databases, potentially constraining the breadth of research insights. Secondly, bibliometric analysis is limited in its ability to evaluate the quality of individual studies. Consequently, although publication and citation metrics provide a broad overview, they may not adequately represent the scientific rigor of the included research.

## 6. Conclusion

This bibliometric analysis systematically examined the scope and evolution of research on mitophagy in osteoarthritis. It highlighted publication trends, collaborative networks, and keyword co-occurrences, identifying research hotspots in areas such as apoptosis, oxidative stress, and mitochondrial dysfunction. Emerging frontiers indicate a growing focus on the role of mTOR signaling and cell death mechanisms in osteoarthritis. This study provides a comprehensive overview that informs future research directions and serves as a valuable reference for scholars and practitioners. The findings enhance our understanding of this evolving field and may have significant implications for clinical approaches to managing osteoarthritis.

## Author contributions

**Conceptualization:** Liping Wang, Dengyan Bai, Xin Ma.

**Data curation:** Liping Wang, Dengyan Bai.

**Formal analysis:** Liping Wang, Xin Ma.

**Writing – original draft:** Liping Wang, Dengyan Bai, Xin Ma, Guan Wang, Zhangbin Luo.

**Writing – review & editing:** Liping Wang, Dengyan Bai, Xin Ma, Guan Wang, Zhangbin Luo.
